# Connecting data science and translational simulation

**DOI:** 10.1186/s41077-026-00410-5

**Published:** 2026-02-17

**Authors:** Victoria Brazil

**Affiliations:** Translational Simulation Collaborative, Faculty of Health Sciences and Medicine, Bond University, Gold Coast, Australia

**Keywords:** Translational simulation, Data science, Healthcare quality improvement

## Abstract

Translational simulation is designed to explore and improve healthcare systems and performance. But this potential can only be realised if simulation activities generate actionable insights, using methods that are efficient and cost effective. Robust data strategies are required, embracing established quality‑improvement (QI) frameworks and the recent applications of artificial intelligence. Data collection, analysis and presentation are the *primary functions* of simulation for quality improvement. This requires translational simulation practitioners to adopt disciplined application of data science, if their diagnostic and interventional simulations are to be translated into tangible gains in healthcare quality and safety.

This article is presented in three parts. First, I review contemporary data science principles in QI and emerging capabilities for data capture, synthesis, and tailored dissemination of findings. Second, I illustrate these principles through case vignettes drawn from the literature. Third, I synthesise these lessons to extend Nickson’s Input–Process–Output model, offering guidance for data strategy development for translational simulation initiatives. By integrating a rigorous data orientation into the foundational IPO schema, I argue that translational simulation can better realise its potential.

## Introduction

I have championed the application of simulation for healthcare quality improvement [1–3], but admittedly have lacked a consistent approach to data collection, analysis and reporting in my simulation practice. Translational simulation [3] – simulation that is designed to explore healthcare systems and to test planned changes - must yield useful insights, using methods that are efficient and feasible. Data strategies are critical. Exemplar projects have demonstrated how this might be achieved: testing new hospitals [4, 5], finding more efficient workflows [6], improving equipment set ups [7], and exploring relational aspects of care [8]. However, the underpinning data science principles to guide practice have yet to be distilled. Two key factors might help: the data strategies embedded in quality improvement (QI) methodologies, and the application of artificial intelligence (AI) to support effective data collection, analysis and dissemination. In this first-person account as a simulation practitioner embedded in a health service, with more than 10 years’ experience in translational simulation, I aim to share the uncertainty and sense of possibility I feel as I wrestle with these opportunities. I advocate that embracing data science is essential for our use of simulation as an improvement technique.

My training as a clinician and educator does not necessarily prepare me well for managing data for quality improvement purposes, and I expect I’m not alone. Without training or guidance in data science, enthusiastic translational simulation practitioners can fall victim to ‘data deluge’ - generating more data than can be successfully (and efficiently) analysed or actioned. Wisdom may be buried in data if our question is not clear. For example, identifying hundreds of latent safety threats (LSTs) while conducting simulations to evaluate a new hospital is unhelpful if we have no organisational strategy or resources to address those LSTs, nor any approach to rank order their likelihood or significance. Debriefing conversations after a simulation to test a cognitive aid for paediatric seizure management may reveal rich insights, but unhelpful if we fail to record these in our notes or find time to analyse them. Measuring improved ‘door to needle’ time in acute stroke care is unhelpful without statistical methods and expertise to determine whether improvements are significant. Without actively *managing* data, we risk adding to negative perceptions of simulation as a resource intensive exercise that doesn’t generate tangible improvements or actionable insights.

Like many other simulation practitioners, I already embrace data strategies for simulation-based *education.* For example, we routinely collect and analyse data are for educational needs assessment and for program evaluation. In this context, data is used to *improve* our simulation based educational programs or *prove* their effectiveness. By contrast, the conceptual framing of translational simulation – its purpose, process and conceptual foundations – is different to that of simulation-based education or simulation-based research. Whether through diagnostic or interventional functions, translational simulation aims to directly improve quality and safety in healthcare through a systems approach. This demands that we have a new relationship with data science: data collection, analysis, and presentation are the primary functions of translational simulation, to inform healthcare quality improvement.

### What is ‘data science’?

Data science is an interdisciplinary field that involves extracting knowledge and insights from structured and unstructured data through scientific methods, algorithms, and systems [9]. It draws upon techniques from statistics, computer science, and domain-specific knowledge. It aims to identify patterns, predict future behaviour or phenomena, and hence support complex problem-solving and decision-making. Examples of data science applications include predicting consumer behaviour, optimizing supply chains, and analysing medical records to improve healthcare outcomes. Data science embraces a breadth of data types, including narrative and qualitative data. Importantly, it is a *science*; one that embraces a myriad of techniques but follows core logic and analytical principles. However, the term ‘data science’ is more than technical, logical data practice. Good data practice helps us measure what matters; data science helps us work out what matters in the first place. It requires value judgements about problem definition and what to measure, combining diverse forms of evidence, and supporting sense-making in complex clinical environments.

Despite reading about data science and quality improvement tools, and experimenting with AI tools to support data capture and analysis, I’m not sure how these should be applied to my translational simulation practice. I need guidance, and that guidance needs to be context agnostic. My translational simulation practice is applied to diverse challenges in my health service: preparing for the opening of new healthcare spaces, exploring interdisciplinary team interfaces, and testing new clinical protocols and cognitive aids. Hence, principles are preferable to prescriptive recipes. Scaffolding these elements into Nickson’s Input Process Output framework for translational simulation [10] may be a logical approach. That framework already embraces an ‘action’ orientation, and distils principles for translational simulation design, delivery and data collection.

In addressing my need, and hopefully that of others, this article will continue in three parts. The first is a deep dive into data science, focused on the quality improvement context. The second presents a series of case examples drawn from the literature to illustrate practical application within translational simulation contexts. The third will draw upon that knowledge and its application to refine Nickson’s Input-Process-Output (IPO) model [10] to more comprehensively guide data strategies for translational simulation. I’ll conclude with some hopes and hesitations for future work in this area.

### Data science and measurement in quality improvement

The challenges of data collection, analysis and dissemination are not new for healthcare quality improvement. Since Plan-Do-Study-Act (PDSA) cycles were first popularised, improvement practitioners have had to decide what to measure and how, and whether a change has led to (measured) improvement [11]. Experience over the last 30 years has matured the paradigms, frameworks and specific tools used by the quality improvement community [12]. Effective data strategies within quality improvement are multifaceted, embrace qualitative and quantitative sources, and employ myriad analytic techniques [13]. Shah outlines the necessity of using a family of measures, including outcome, process, and balancing measures [14]. He highlights time series analysis, particularly using small amounts of data collected and displayed frequently, as the gold standard for monitoring improvements. This aligns with the ‘intervention logic’ inherent in most healthcare improvement efforts. i.e. we measure a baseline state, introduce and intervention and then measure again to see if improvement has occurred.

There are alternative paradigms for improvement in healthcare, and associated data strategies. Liberati described a ‘context logic’ paradigm for improvement when reporting an ethnographic study of a high performing maternity unit in the United Kingdom [15]. A ‘context logic’ involves “*identifying the features of particular environments (such as organisational structures*,* processes*,* behaviours*,* practices*,* and values) that contribute to safety*” [15]. This paradigm may be more appropriate for translational simulation activities that are *diagnostic*, i.e. exploring (and collecting data about) work environments and the people in them before ‘fixing’. Simulation practitioners often occupy a unique position in health systems - as clinicians, educators, and improvers. This allows recognition of patterns and problems that may not be visible in formal datasets. Influenced by the work of Dixon-Woods and others [15], I realise that data strategies are not simply “applied” to contexts; they are formed within them. Embracing data science for improvement requires both methodological discipline and contextual sensitivity.

### How does this apply to translational simulation practice?

Robust use of data ensures that translational simulation is purposeful, measured, and accountable. I use ‘data science’ to also signal something broader: a disciplined approach to framing the right questions, integrating multiple sources of qualitative and quantitative data, analysing them with appropriate methods, and iteratively learning in complex systems. Data science is a field of robust debates, risks, and value judgements, and there are competing visions of what ‘good’ looks like. Translational simulation is context-sensitive and oriented to organisational learning in complex environments. Hence, the integration of data science surfaces real tensions in translational simulation practice - trust vs. opacity, usefulness vs. burden, power and interests - in how we collect data and what we do with it.

Re-prosecuting the voluminous literature relating to data science in quality improvement is beyond the remit of this article. My refocus on data science within translational simulation practice does not manifest as a long list of QI tools. The outcome is simpler: becoming more rigorous in the question we are trying to answer with our translational simulation programs or projects - whether diagnostic or interventional – and more proactive in thinking about how that question can be answered. Choices about QI methodologies, data collection, and analysis are profoundly easier if the question is clear.

One recent example from my practice involved using simulation to test readiness prior to the opening of a redesigned triage area in an emergency department. Previously I might have simply engaged relevant staff in managing simulated emergencies in the new space and sought broad, unstructured feedback, and felt reassured that staff had been orientated to the new space. However, with my newfound ‘data discipline’, I narrowed my question: ‘*do staff have the equipment*,* environment*,* and call systems to manage a patient with either a medical or behavioural emergency in the new space*?’. This enabled more careful design of simple scenarios, delivery using cardboard cutouts as patients, structured debriefings using those pre-identified issues, and analysis of those debriefing transcripts by AI tools prompted to conduct analysis according to that question. This ‘question discipline’ should be a core feature of translational simulation. Data science is not merely a means of tracking improvement, but offers a mindset - identifying puzzles, anomalies, and misalignments that are central to improvement efforts.

### How might Artificial Intelligence help?

In 2025, discussions about data science inevitably turn to generative artificial intelligence (AI) and machine learning. AI supported tools can tame copious and unruly data, and may help integrate data science into all stages of planning, delivery and data collection for translational simulation. Artificial intelligence (AI) and machine learning (ML) might help process large datasets efficiently and provide insights that were previously difficult to obtain. Large language models (LLMs) (e.g. ChatGPT, Perplexity etc.) might offer support for project planning, scenario/ session design, data collection and analysis, and production of reports. These applications remain in their infancy, and harnessing powerful AI tools for data management starts with a foundational understanding of data science and its application within translational simulation.

### Practical examples of data science in translational simulation

Given the breadth of the discussion so far, it might be helpful to illustrate the application of data science to translational simulation through examples. What follows are three published examples, selected to illustrate the variation in scale and nature of data being collected and analysed from translational simulation activities.

#### A departmental project using quantitative data

Butler et al. used translational simulation to decrease the time to obtain equipment needed for adult advanced airway management by nurses and physicians in a rural emergency department (ED) in British Columbia, Canada [7]. Using a quality improvement framework, Plan-Do-Study-Act (PDSA) cycles were used to evaluate the impact of practice changes that had been identified and trialled in a translational simulation program. Changes included the introduction and subsequent modification of an airway cart, to support practitioners’ equipment retrieval. The team achieved a 76% decrease in the time to obtain airway equipment and increased provider comfort at the end of their study period [7].

Considering this work through the lens of data science and translational simulation, I make four observations. First, the question to be answered was clear: could time to retrieve airway equipment be shortened through iterative process interventions? Second, the PDSA cycle framework provided a conceptual scaffold for the improvement process. Third, the data strategy was embedded from the planning stage: quantitative data was collected and analysed using Shewhart charts constructed in Microsoft Excel [16]. Fourth, translational simulation served both an exploratory function and as a test bed in which to trial improvements [3].

#### Transfusion policy redesign using Failure Mode and Effects Analysis (FMEA) methodology

Dube et al. describe using system-focused simulation to identify system issues in a large healthcare organization’s transfusion policy redesign [17]. Existing patient safety data identified high risk issues in transfusion practice within the health authority: administering (i.e. the nursing role from cross-checking blood to actual transfusion in the clinical area), and preparing, dispensing or packaging blood and blood products in the lab. This data was used to inform simulations designed to explore system and process challenges related to blood transfusion and opportunities to improve the existing policy. Debriefings were conducted using a modified PEARLs approach that focused the conversation on systems and process issues. A Failure Mode and Effects Analysis (FMEA) [18] was used to apply a risk score to the findings from the simulation user feedback to inform policy redesign. FMEA scoring is calculated according to the (i) severity or impact of the failure, (ii) frequency of the failure and (iii) the likelihood of detection resulting in an overall ‘criticality score’. As a result of this work, the blood administration policy and procedure were revised, the laboratory physical space was modified, and multiple work processes were changed.

Considering this work through the lens of data science and translational simulation, I make four observations. First, data was used to *inform* problem identification and simulation design. Second, an explicit problem identification and data strategy informed an effective structure for debriefings that enabled rich data collection from frontline clinicians. Third, the large volumes of qualitative data were able to be analysed using a robust methodology – FMEA scoring – that translated the data into actionable insights for the organisation. Fourth, translational simulation served both an exploratory function and as a test bed in which to trial improvements.

#### Translational simulation for the opening of a new facility: harnessing qualitative data

Oliver et al. used translational simulation as a component of the change management process in supporting the transition to a new hospital in Edinburgh, United Kingdom [19]. Their simulation approach was guided by, and reported according to, Nickson et al.’s IPO model [10]. Simulation design was informed by understanding the key staff concerns surrounding the realities of relocation, through questionnaires, surveys and informal site visits with staff teams. Latent safety threats were identified during the simulation and debriefing phases of the project, and ‘strategically disseminated’ to clinical teams and hospital leadership. The author team were also interested in how their translational simulation programme impacted the *change experience* of healthcare professionals during the hospital relocation. Answering this question involved interviews with staff, and Kolbe et al.’s ‘What’s the Headline in your Mind Right Now?’ approach [20], requesting that participants wrote brief thoughts on Post-It™ notes during the simulation sessions. Analysis of this qualitative data revealed insights about how translational simulation enabled participation, sensemaking and building team relationships that smoothed the experience of transition.

Considering this work through the lens of data science and translational simulation, I make three observations. First, multiple sources of data were used to inform problem definition, specific scenario designs and choice of debriefing model. Second, qualitative data was equally, if not more, important than quantitative measures to support improvement in this context. Third, data collected from simulations supported two purposes: (1) local and contextual identification of latent safety threats, and (2) scholarly outputs in the form of deeper understanding of the processes by which principles of translational simulation can support change management.

Reflecting on these examples and my own translational simulation practice, I aim to extract general principles from the context specific projects, and expand upon them, to offer practical guidance for translational simulation practitioners. Data strategies need to be embedded and intertwined at every stage of translational simulation activities. Data collection and analysis cannot be an afterthought, nor can it be haphazard. Clear problem identification, proactive data strategy development, and effective match of data methods to the context (including feasibility) are critical. While contexts may vary, these principles may be made practical through a guiding conceptual model that will be offered in the next section.

### Building on the IPO model: data, data, data

**Fig. 1:**
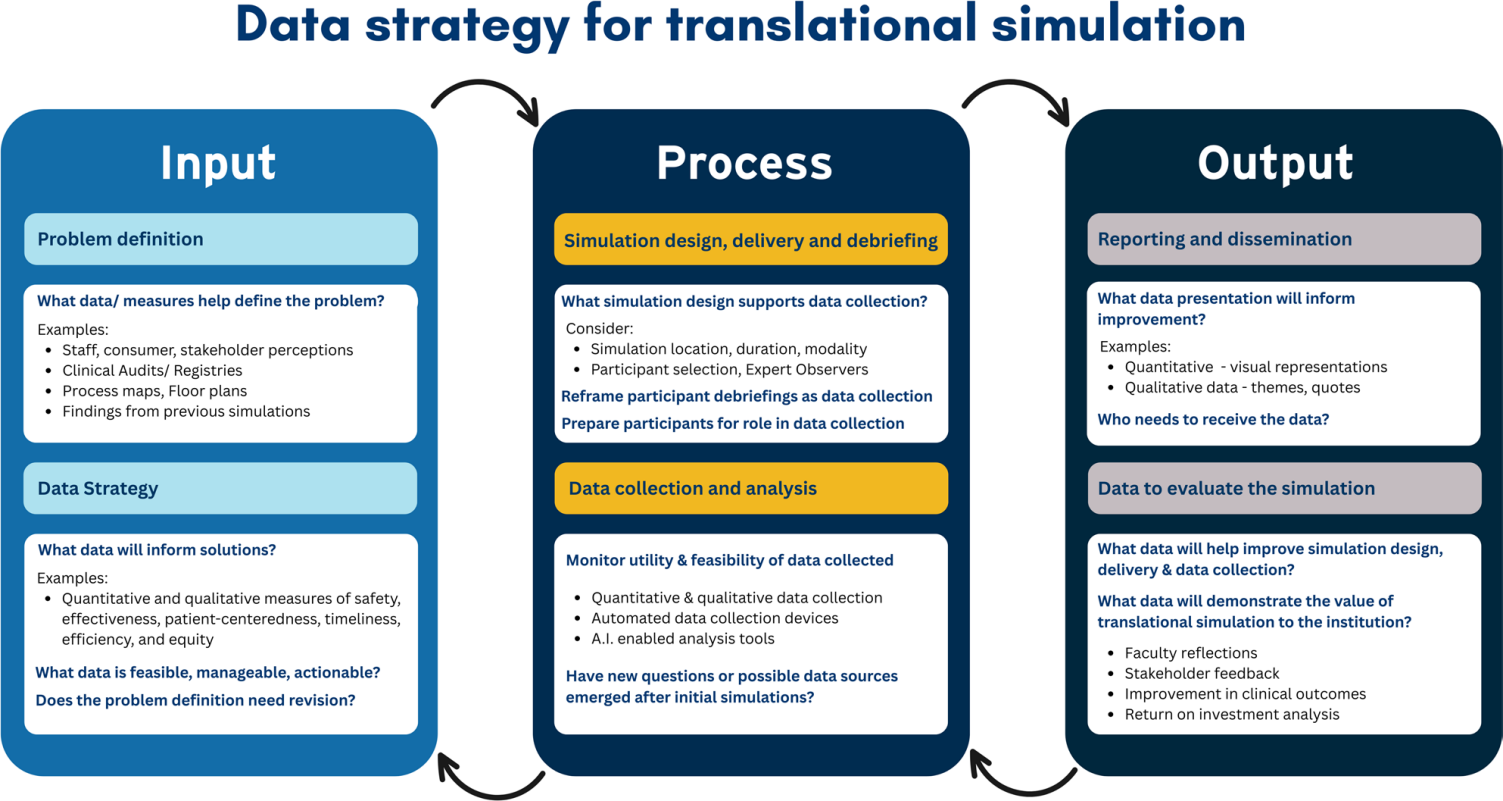
Data strategy within the IPO Model. 10.5281/zenodo.17248152 Creative Commons licence CC BY 4.0. *Adapted from Nickson et al. Translational Simulation: From Description to Action*,* Advances in Simulation. 2021* [10] 10.1186/s41077-021-00160-6

Nickson’s Input-Process-Output (IPO) model is a foundational framework that has provided a valuable road-map for how simulation can drive healthcare improvement. It provides a scaffold upon which to explicitly plan and execute a data strategy for translational simulation activities. Conceptual models must be iterative, adaptable, and reflective of shifting practice environments [21]. This is not an abandonment of foundational thinking, but rather a scholarly imperative to refine relevance, inclusivity, and explanatory power in light of contemporary challenges [22]. In the outline that follows, I integrate data science perspectives into Nickson’s model, to expand its scope and utility, while preserving its conceptual integrity. Translational simulation may be framed as problem-finding practice, in which questions are the anchor for data strategy. Using the scaffolding of the IPO model, Fig. 1 illustrates a series of key questions that guide practitioners to focus on the data strategy embedded within their overall project [23].

#### Input

The Input phase requires a disciplined focus on problem definition, informed by data, and refined by the rigorous planning of a data strategy to address the problem. Simulation practitioners working in health service contexts will have limitations on their capacity to collect and analyse data from simulations. Not all data will generate actionable insights to inform improvements. Data gleaned from simulations may not be cost effective or add value to existing quality improvement methods for the problem. Hence the focus of the Input stage is threefold: (1) *what data can inform problem identification*, and (2) *what data collection and analysis will be aligned and feasible* to address that problem through simulation, and (3) *does the problem need to be revisited in the light of that?*. Nickson’s article provides a helpful table of data sources for the former question: audits, clinical incident data, quality benchmarking, stakeholder interviews, capital works designs and more [10]. Addressing the second and third questions draws these data considerations into the earliest phases of translational simulation planning.

A disciplined data strategy also rests on relationships. Decisions about what to measure and why should be shaped through meaningful engagement with those who experience the problem - clinicians, leaders, patients, and organisational partners. As Roussin et al. [24] describes, this requires more than securing “buy-in”; it involves co-producing the problem definition, understanding stakeholder pain points, and recognising that institutional priorities may not always align perfectly with frontline or patient needs.

#### Process

The Process phase requires thoughtful simulation design to focus on the issue to be addressed, engage participants, and collect useful data. A comprehensive list of specific data collection tools are offered in Nickson’s Table 2, including quantitative measures of performance, structured observer measurement tools, and participant narrative reflections in debriefing conversations [10]. AI tools can improve the ability to collect and analyse larger amounts of data - qualitative and quantitative - than would previously have been practical for many translational simulation practitioners [25]. However, AI-driven data analysis remains vulnerable to error. Data of dubious quality, structural biases embedded in the training corpus, and the opacity of “black-box” algorithms can compromise the reliability of its conclusions [25, 26]. ‘Hallucinations’ - when an LLM generates output that appears plausible but is factually incorrect or entirely fabricated – are well documented [27, 28], and introduce significant risk if AI tools are used without careful human oversight.

Important data considerations for the Process phase include preparing simulation participants for their role as sources (and interpreters) of data, and re-framing debriefing conversations as ‘data collection’. This re-framing is important for participants who might have previously expected to *receive* feedback after participating in educationally simulations but instead are now asked to *provide* feedback on the adequacy of systems or workflows and suggestions for improvement. Modified debriefing structures and tools for this purpose have been described and widely used [29, 30]. The second part of the Process phase recognises the dynamic and iterative nature of translational simulations. Practitioners should ask whether data collection choices should be refined after initial simulations, or, even more fundamentally, whether new questions or possible data sources emerged?

#### Output

The Output phase requires data collection and analysis to be translated to actionable insights that are provided to the right stakeholders to inform improvement. Nickson’s article (in his *Table 3*) provides helpful guidance for dissemination methods and audiences [10]. To enhance impact, reports should use effective data visualisation tools for quantitative and qualitative data [31]. Recently developed AI tools have brought sophisticated data visualisation capabilities to everyday simulation practitioners [32]. Data presentation should also be aligned with local institutional quality improvement language and risk management policies.

The second part of the Output phase shifts focus to the translational simulation design and delivery process itself; what data offers insights into how the simulation can be improved, and/or what data demonstrates the value of the simulation process. There is an overlap but also some clear distinctions between this data and that which is presented to inform the healthcare improvement target. For example, faculty insights into whether modality choices were appropriate or whether debriefs were appropriately structured are themselves sources of data that can inform our internal simulation program quality assurance. Ideally these should be systematically collected and analysed, together with data on the cost and resource allocation for simulation activities. Likewise, most simulation practitioners are acutely aware of the need to demonstrate the value of their work to those that fund them. The benefits of translational stimulation activities may be broader than simply the addressing the identified problem, and more diverse sources of data may demonstrate a broader spectrum of value to the organisation [33].

### Data science: hope and hesitation

Data is never neutral, and neither are the systems that encourage us to collect, analyse, and report it. Data may be seductive – conferring a disproportionate sense of legitimacy and sophistication. Data science should not equate to more performative work: dashboards, process control charts, or AI-generated summaries that demonstrate effort rather than insight. My challenge is not simply “*how do we do better data science?*” but “*what different or better thinking does data science allow us to do?*”. Data strategies should support simulation teams and stakeholders to see their systems more clearly, and provide actionable insights to their health services. They should help identify what cannot yet be measured, as much as they help quantify what can.

## Conclusion

Without thoughtful attention to data science at all phases of translational simulation, we risk our work being data-rich but insight-poor. But, empowered by data strategies embedded in quality improvement, our capacity to translate simulation-based organisational learning to real world practice has never looked brighter. Building on the IPO model, I encourage translational simulation practitioners to embrace a renewed focus on data collection, analysis and reporting - to identify problems to be addressed and to realise the promise of simulation as an improvement technique.

## Data Availability

No datasets were generated or analysed during the current study.

## Abbreviations

AI: Artificial Intelligence
QI: Quality Improvement
LST: Latent Safety Threat
IPO: Input–Process–Output
ED: Emergency Department
PDSA: Plan–Do–Study–Act
FMEA: Failure Mode and Effects Analysis
PEARLs: Promoting Excellence and Reflective Learning in Simulation
ML: Machine learning
LLM: Large Language model
